# Smoking is Associated With Impaired Long-term Quality of Life in Elderly People: A 22-year Cohort Study in NIPPON-DATA 90

**DOI:** 10.2188/jea.JE20220226

**Published:** 2024-06-05

**Authors:** Yiwei Liu, Tomonori Okamura, Aya Hirata, Yasunori Sato, Takehito Hayakawa, Aya Kadota, Keiko Kondo, Takayoshi Ohkubo, Katsuyuki Miura, Akira Okayama, Hirotsugu Ueshima

**Affiliations:** 1 Department of Preventive Medicine and Public Health, Keio University School of Medicine, Tokyo, Japan; 2 Research Center for Social Studies of Health and Community Ritsumeikan University, Kyoto, Japan; 3 Department of Public Health, Shiga University of Medical Science, Shiga, Japan; 4 Department of Hygiene and Public Health, Teikyo University School of Medicine, Tokyo, Japan; 5 Research Institute of Strategy for Prevention, Tokyo, Japan; 6 NCD Epidemiology Research Center, Shiga University of Medical Science, Shiga, Japan

**Keywords:** quality of life, smoking, tobacco control, cohort study

## Abstract

**Background:**

Whether smoking is associated with worse quality of life (QoL) or not is relatively controversial. The current study is to investigate the relationship between smoking and subjective QoL in a long cohort study.

**Methods:**

The NIPPON DATA 90 project collected 8,383 community residents in 300 randomly selected areas as baseline data in 1990, administered four follow-up QoL surveys, and evaluated mortality statistics. We conducted multinomial logistic regression analysis to compare past smokers and current smokers to never smokers, with impaired QoL and mortality as outcomes.

**Results:**

In four follow-ups, QoL data was collected from 2,035, 2,252, 2,522, and 3,280 participants in 1995, 2000, 2005, and 2012, respectively. In the 1995 follow-up, current smoking at baseline was not associated with worse QoL. In 2000 and 2005 follow-ups, smoking was significantly associated with worse QoL (odds ratio [OR] 2.1; 95% confidence interval [CI], 1.33–3.36 and OR 2.29; 95% CI, 1.38–3.80, respectively). In the 2012 follow-up, smoking was not associated with QoL. Sensitivity analysis did not change the result significantly.

**Conclusion:**

In this study we found that baseline smoking was associated with worse QoL in long-follow-up.

## INTRODUCTION

Evidence suggests that smoking is associated with worse health-related quality of life (QoL) among different populations.^1^^–^^4^ The United States Food and Drug Administration (FDA) decided to discount graphic warnings by 70 percent to offset the perceived loss in pleasure experienced by smokers when they give up their habit.^5^ However, there has been a debate regarding whether we should discount the short-term quality-adjusted life years (QALYs) associated with the tobacco ban.^6^ Since tobacco control policy primarily focuses on long-term health-related issues, it is important to consider the impact on long-term QoL as well. For instance, regardless of health issue, does smoking itself affect QoL in the long term? If so, we need to adjust QALYs for time period.

Nevertheless, only a limited number of studies have investigated whether smoking is associated with long-term impaired QoL compared to individuals who have never smoked. Additionally, it remains unclear whether smoking influences QoL beyond health-related issues. To arrive at a more definitive conclusion, the current study aims to examine the relationship between smoking and long-term QoL while addressing potential biases related to mortality right censoring effects. This will involve conducting robustness checks to ensure the validity of the findings. The findings will contribute to the existing academic literature and inform future tobacco control policies and public health interventions aimed at precisely improving overall QoL in the long term.

## METHODS

### Participants

**Figure 1.**
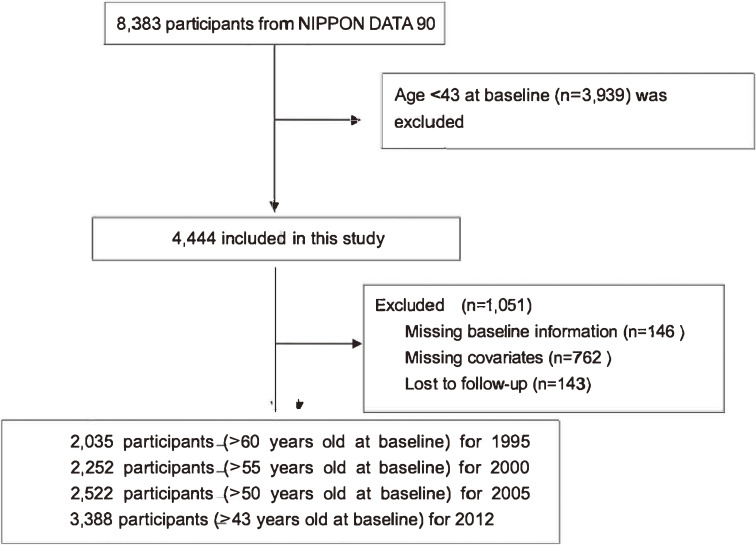
Flow chart for study inclusion. Participants were 4,444 community residents aged 43 and older in 300 randomly selected areas; 3,393 participants without missing information were followed until 2012.

The cohort studies of the National Survey on Circulatory Disorders, Japan, are referred to as the National Integrated Project for Prospective Observation of Non-communicable Disease And its Trends in the Aged (NIPPON DATA) 90. This project was to investigate risk factor for non-communicable disease in senior Japanese population. The details of this cohort have been reported previously^7^; in brief, a total of 8,383 community residents (3,504 men and 4,879 women) aged >30 years old were recruited by local public health centers in 300 randomly selected areas and participated in the survey in 1990, with no other inclusion criteria deployed. Participants aged 43 years and older at the baseline survey (*n* = 3,422) were selected for the present study, most of them followed up to 2012 (missing follow up excluded, *n* = 34). In these participants, we investigated their QoL when they reached 65 years old at the four follow-up surveys. Accordingly, 2,035, 2,252, 2,522, and 3,388 participants without missing data in 1995, 2000, 2005, and 2012 respectively, were included in the present study (Figure 1).

### Ethical approval

The current study was conducted in accordance with the ethical guidelines of Shiga University of Medical Science (R2005-021) and Keio University School of Medicine (2018-0108).

### Exposure and covariates

For smoking status, we defined “never, past, and current” smokers as binary variable (yes or no) from survey questions: did you never/almost never smoke? Did you smoke and quit now? Do you currently smoke? Those answered yes would be classified as never, past, and current smokers, respectively. For other variables, except QoL and mortality, all data (including smoking status) were collected from baseline by local public health center and no follow-up data was available. In all analyses, when we adjusted covariates or showed characteristics in tables, we were using baseline covariates data. Smoking status was treated as three categories (never, past, and current smokers) in primary analysis, and in sensitivity analysis, we treated exposure as two categories (past + current = smoker, never = non-smoker). A previous study from NIPPON DATA90 has shown how other covariates were measured, particularly, drinking status were counted as drinker (≥1 drink/week in average) or non-drink (<1 drink/week in average).^7^

### Outcome measurement

Our primary outcome was impaired QoL as a binary outcome, defined as any QoL items showing equal or worse than neutral option, including life meaning, life satisfaction, and happiness, using a questionnaire that has been published previously.^8^ Public health nurses collected information about QoL principally through face-to-face or telephone interviews. Participants were asked about three QoL items, including life meaning (do you think your current living is meaningful? Options: yes, neutral, no), life satisfaction (would you satisfy your overall current status? Options: yes, kind of, neutral, maybe not, no), and well-being (can you feel happiness in your current living? Options: yes, kind of, neutral, maybe not, no).

Our secondary outcome was mortality. Vital statistics for determining causes of death were obtained from the Management and Coordination Agency, Government of Japan. The underlying causes of death for the National Vital Statistics were coded according to the 9^th^ International Classification of Disease (ICD-9) until 1994 and the 10th International Classification of Disease (ICD-10) from 1995. Details of these classifications have previously been described.^9^

### Statistical analysis

We used multinomial logistic regression analysis to compare past smokers and current smokers to never smokers, with impaired QoL and mortality as outcomes. We adjusted age, gender, alcohol drinking, systolic blood pressure, diastolic blood pressure, total cholesterol, blood glucose, and serum albumin as covariates in the full model.

### Sensitivity analysis

First, due to competitive risk as mortality to QoL measurement, we conducted a pattern mixture model (PMM; using R package missingHE) to evaluate the impact of censoring issue. Since PMM requires no missingness, we imputed all missing values (none of them over 10%) using R package mice.^10^ In this analysis, we merged the past smoker with the current smoker group due to limited statistical power. Patient strata (smoking) or censor (mortality) patterns were defined by the time of patient censor from the QoL assessments. The PMM included a term describing the interaction between smoking status, time and stratum group, and other covariates (including age, gender, systolic blood pressure, diastolic blood pressure, cardiovascular disease [CVD] treatment, drinking, local population size, HbA1c, total cholesterol, serum albumin and triglyceride) were fit to the data for the primary outcome. Second, we converted exposure into smoking years as a continuous variable and conducted the same analysis as for the primary outcome to see if this would affect our result. All analysis was conducted in R 4.0.1 (R Foundation for Statistical Computing, Vienna, Austria).

## RESULTS

In four follow-ups, QoL data was collected from 2,035, 2,252, 2,522, and 3,280 participants, in 1995, 2000, 2005, and 2012, respectively. Their baseline characteristics (not stratified by follow-up) are shown in Table 1 (see eTable 1, eTable 2, eTable 3 and eTable 4 for the followers in each year). Multinomial logistic regression was used to analyze impaired QoL in 1995, 2000, 2005, and 2012. The results showed that current smoking was associated with impaired QoL in the 2000 and 2005 follow-ups (odds ratio [OR] 2.11; 95% confidence interval [CI], 1.33–3.36 and OR 2.29; 95% CI, 1.38–3.80, respectively), but no significant association was found in current smokers for the 1995 and 2012 follow-ups. Additionally, no significant association was found for past smokers (Table 2). In the analysis of the secondary outcome, we found a significant association with higher mortality risk in current smokers (OR 1.71; 95% CI, 1.26–2.33, OR 3.57; 95% CI, 2.56–4.98, and OR 3.07; 95% CI, 2.27–4.13 for 2000, 2005, and 2012, respectively). In the PMM model, most QoL and mortality were significantly associated with smoking, except life satisfaction and happiness in the 2012 follow-up (Table 3). When we converted exposure into a continuous variable (smoking years), the main result did not change significantly (data not shown).

**Table 1.** Baseline characteristics for total participants

	Current Smoking (*n* = 976, 28%)	Past Smoking (*n* = 394, 12%)	Never Smoking (*n* = 2,023, 60%)
Age, years, mean (SD)	61 (10)	66 (10)	64 (11)
Gender, male, %	85%	89%	32%
BMI, kg/m^2^, mean (SD)	23 (3)	23 (3)	23 (3)
Systolic blood pressure, mm Hg, mean (SD)	144 (21)	145 (20)	143 (20)
Diastolic blood pressure, mm Hg, mean (SD)	80 (12)	84 (12)	82 (12)
CVD treatment,^a^ %	23%	34%	30%
Drinking, %	45%	46%	17%
Salt preference, % prefer salty	61%	54%	49%
HbA1c, mmol/L, mean (SD)	5 (0.7)	5 (0.7)	5 (0.7)
Total cholesterol, mg/dL, mean (SD)	205 (39)	202 (38)	198 (38)
HDL-C, mg/dL, mean (SD)	51 (15)	51 (16)	53 (16)
Serum albumin, mg/dL, mean (SD)	4.4 (0.3)	4.3 (0.3)	4.4 (0.3)
Triglyceride, mg/dL, median [interquartile range]	144 [83–171]	142 [83–177]	136 [80–164]

BMI, body mass index; CVD, cardiovascular disease; HDL-C, high density lipoprotein-cholesterol; HbA1c, glycated hemoglobin; SD, standard deviation.

^a^CVD related treatment stands for receiving any blood pressure/glucose/lipid lowering medication therapy.

**Table 2.** Multinominal logistic regression model for impaired QOL and mortality outcome for 1995, 2000, 2005, 2012 follow-ups

**Multinominal logistic regression model for impaired QOL in 4 follow-ups**				
(reference: never smoker)	1995	2000	2005	2012
Never smoker, events/total (%)	79/1,131 (7%)	99/1,261 (8%)	98/1,419 (6%)	117/1,909 (6%)
Past smoker, events/total (%)	23/343 (7%)	24/361 (7%)	18/382 (5%)	16/499 (3%)
Current smoker, events/total (%)	58/561 (10%)	74/630 (12%)	58/721 (8%)	40/980 (4%)
Past smoker, Crude OR (95% CI)	1.00 (0.61–1.62)	1.03 (0.64–1.64)	1.02 (0.60–1.76)	0.81 (0.46–1.42)
Model 1 OR (95% CI)	1.00 (0.62–1.63)	1.01 (0.62–1.62)	0.94 (0.55–1.62)	0.76 (0.42–1.33)
Model 2 OR (95% CI)	0.92 (0.49–1.75)	1.07 (0.60–1.94)	1.06 (0.53–2.13)	0.83 (0.42–1.66)
Current smoker, Crude OR (95% CI)	1.70 (1.19–2.44)	1.93 (1.38–2.68)	1.93 (1.35–2.77)	1.27 (0.85–1.89)
Model 1 OR (95% CI)	1.78 (1.23–2.54)	1.78 (1.23–2.54)	2.11 (1.46–3.04)	1.38 (0.93–2.06)
Model 2 OR (95% CI)	1.75 (0.98–2.73)	2.11 (1.33–3.36)	2.29 (1.38–3.80)	1.52 (0.89–2.59)

**Multinominal logistic regression model for mortality outcome in 4 follow-ups**				
(reference: never smoke)	1995	2000	2005	2012

Never smoker, events/total (%)	158/1,131 (14%)	346/1,261 (27%)	611/1,419 (43%)	1156/1,909 (61%)
Past smoker, events/total (%)	60/343 (18%)	143/361 (40%)	237/382 (62%)	376/499 (75%)
Current smoker, events/total (%)	118/561 (21%)	238/630 (39%)	443/721 (61%)	769/980 (78%)
Past smoker, Crude OR (95% CI)	1.30 (0.94–1.81)	1.73 (1.35–2.22)^***^	2.16 (1.69–2.74)^***^	1.99 (1.57–2.52)^***^
Model 1 OR (95% CI)	1.31 (0.94–1.82)	1.73 (1.30–2.29)^***^	1.92 (1.43–2.57)^***^	1.72 (1.32–2.23)^***^
Model 2 OR (95% CI)	1.08 (0.69–1.68)	1.80 (1.27–2.55)^***^	2.04 (1.39–3.01)^**^	1.56 (1.11–2.21)^**^
Current smoker, Crude OR (95% CI)	1.73 (1.33–2.26)^***^	1.93 (1.38–2.68)^***^	2.34 (1.93–2.85)^***^	2.02 (1.48–2.75)^***^
Model 1 OR (95% CI)	1.93 (1.47–2.53)^***^	2.03 (1.46–2.83)^***^	4.02 (3.61–5.11)^***^	3.65 (2.93–4.54)^***^
Model 2 OR (95% CI)	1.43 (0.97–2.11)	1.71 (1.26–2.33)^***^	3.57 (2.56–4.98)^***^	3.07 (2.27–4.13)^***^

CI, confidence interval; OR, odds ratio; QOL, quality of life.

^*^P < 0.05, ^**^P < 0.01, ^***^P < 0.001.

Model 1 adjusted for age, model 2 adjusted for adjusted by age, gender, alcohol drinking, systolic blood pressure, diastolic blood pressure, salt preference, total cholesterol, blood glucose and serum albumin.

**Table 3.** Pattern mixture model for QOL and smoking

	Smoking mean effect on QoL, mean (SD)	*P* value	Smoking mean effect on mortality, mean (SD)	*P* value
Life meaning 2012	0.05 (0.06)	0.006	0.12 (0.02)	<0.001
Life satisfaction 2012	0.04 (0.11)	0.138	0.18 (0.02)	<0.001
Happiness 2012	0.04 (0.09)	0.072	0.18 (0.02)	<0.001
Life meaning 2005	0.12 (0.05)	<0.001	0.14 (0.02)	<0.001
Life satisfaction 2005	0.03 (0.01)	0.003	0.14 (0.02)	<0.001
Happiness 2005	0.02 (0.01)	0.006	0.14 (0.02)	<0.001
Life meaning 2000	0.03 (0.05)	0.044	0.12 (0.02)	<0.001
Life satisfaction 2000	0.01 (0.01)	0.025	0.13 (0.02)	<0.001
Happiness 2000	0.03 (0.01)	0.017	0.13 (0.02)	<0.001
Life meaning 1995	0.02 (0.01)	0.060	0.04 (0.02)	0.008
Life satisfaction 1995	0.03 (0.01)	0.023	0.04 (0.02)	0.008
Happiness 1995	0.02 (0.01)	0.031	0.04 (0.02)	0.007

CI, confidence interval; OR, odds ratio; QOL, quality of life.

Model adjusted by age, gender, alcohol drinking, systolic blood pressure, diastolic blood pressure, salt preference, total cholesterol, blood glucose and serum albumin.

## DISCUSSION

In this study, we observed an association between current smoking and worse QoL in the 10- and 15-year follow-ups. However, the results were only marginally significant (*P* < 0.1) in the 5-year follow-up, and no association was found in the 22-year follow-up. To ensure the robustness of our findings, we conducted a PMM analysis. This analysis revealed that the 22-year follow-up result showed a significant association with life meaning but not with life satisfaction and happiness. This model allowed us to correct for bias from non-randomly selected samples, such as participants who died and were unable to participate in our QoL survey. We speculate that the lack of association in the 22-year follow-up may be attributed to right censoring effect, as smoking is known to increase mortality risk. Since follow-up data on health-related outcomes are not available, we can only assume that the heterogeneity in results between follow-ups was due to general health issues.

In previous psychological studies, most of the studies were trying to demonstrate that smoking cessation is not associated with a net loss of pleasure in less than 5 years,^11^ except one with a 26-year follow-up.^1^ Furthermore, in these studies, a key potential bias was not well studied—selection bias could be caused by smoking-contributed mortality (loss to follow-up). As a previous study found out that current smokers tend to have higher QoL score in cancer survivors, which might largely due to the “illusion” from selection bias as mentioned in this study.^12^ In fact, our result showed that in the very long term (∼30 years), baseline smoking status was not significantly associated with QoL (in 2012 follow-up), if we did not correct this bias with the PMM model. One study was trying to address this issue using two-stage least square (2SLS) with endogenous instrumental variable, but this was a cross-sectional study without follow-up.^13^ Such cross-sectional designs suffer from reverse causality issues. Since previous study had already found out that worse QoL is associated with smoking initiation,^14^ this bias could be serious.

Other researchers found that continuous smoking behavior was not a simple ‘pleasure gain’ for all smokers, because some of them gained ‘enjoyment’ and others suffered ‘relief from craving’,^15^^,^^16^ for those suffering from craving would have much worse QoL. Combining these findings with our study, we assume that, in the general smoker population, tobacco control policy might elevate overall QoL for smokers in the long term. The FDA’s tobacco policy regulation (Order 12866) reduced the expected benefits of its tobacco-control policies by 70% to account for the decrease in life satisfaction. However, even if we disregard the impact of health-related outcomes, our findings suggest that this trend could be mitigated, or even reversed in the long term.

Our study has some limitations, the most important of which was the lack of smoking data during follow-ups: we do not know the participants’ smoking status after baseline collection. However, this study was designed to establish baseline smoking in relation to long-term QoL. PMM analysis would allow us to correct bias from non-randomly selected samples—in our case, those who died would not be able to participate in our QoL survey. Although no strict causal relationship could be found, our results demonstrated that smoking cessation is not associated with long-term positive QoL. Another major limitation was that we did not have socioeconomic and mental health data for individuals. Previous studies have demonstrated that this factor is associated with QoL.^17^ Although we tried to adjust for local population size, we did not include it in the final analysis because such ecological data might induce severe bias.

### Conclusion

Overall, we conducted an analysis to evaluate the effect of smoking on long-term QoL. Our result found that smoking is associated with worse long-term QoL for participants over 45 years old, though smoking is harmful even ignoring its health-related effects.

## SUPPLEMENTARY MATERIAL

eTable 1. Baseline characteristics for 1995 follow-up participants

eTable 2. Baseline characteristics for 2000 follow-up participants

eTable 3. Baseline characteristics for 2005 follow-up participants

eTable 4. Baseline characteristics for 2012 follow-up participants
