# Supplementary material for: Smoking is Associated With Impaired Long-term Quality of Life in Elderly People: A 22-year Cohort Study in NIPPON-DATA 90
Source: J Epidemiol. 2024 Jun 5;34(6):265–9. doi: 10.2188/jea.JE20220226 (PMC11078595; doi:10.2188/jea.JE20220226)
Supplement: Supplementary file 1 [file je-34-265-s001.pdf]

eTable 1. Baseline characteristics for 1995 follow-up participants

	Never smoking	Past smoking	Current smoking
n	1,637	454	696
Age, years, mean (SD)	69 (7)	69 (7)	68 (6)
Gender, male, %	14%	87%	82%
BMI, kg/m^2, mean (SD)	23 (3)	22 (3)	22 (3)
Systolic Blood Pressure, mm Hg, mean (SD)	146 (20)	146 (20)	147 (22)
CVD treatment[a]	36%	37%	27%
Drinking	8%	41%	47%
Salt preference, % prefer salty	47%	53%	59%
HbA1c, mmol/L, mean (SD)	5.1 (0.8)	5.1 (0.7)	5.2 (0.8)
Total cholesterol, mg/dL, mean (SD)	215 (40)	202 (39)	197 (39)
HDL-C, mg/dL, mean (SD)	53 (15)	51 (16)	50 (16)
Serum albumin, mg/dL, mean (SD)	4.3 (0.3)	4.3 (0.3)	4.3 (0.3)
Triglyceride, mg/dL, mean (SD)	135 (79)	143 (91)	139 (98)
Life meaning, % impaired	5.1%	3.8%	7.2%
Satisfaction, % impaired	2.3%	2.3%	3.9%
Well-being, % impaired	1.3%	0.7%	1.8%

BMI, body mass index; CVD, cardiovascular disease; HDL-C, high density lipoprotein-cholesterol; HbA1c, glycated hemoglobin; SD, standard deviation.

[a] CVD related treatment stands for receiving any blood pressure/glucose/lipid lowering medication therapy.

eTable 2. Baseline characteristics for 2000 follow-up participants

	Never smoking	Past smoking	Current smoking
n	1,261	361	630
Age, years, mean (SD)	63 (9)	65 (9)	63 (8)
Gender, male, %	16%	88%	83%
BMI, kg/m^2, mean (SD)	23 (3)	23 (3)	23 (3)
Systolic Blood Pressure, mm Hg, mean (SD)	142 (20)	144 (20)	144 (21)
CVD treatment, %[a]	28%	33%	23%
Drinking, %	9%	45%	52%
Salt preference, % prefer salty	47%	52%	63%
HbA1c, mmol/L, mean (SD)	5.1 (0.8)	5.1 (0.8)	5.2 (0.8)
Total cholesterol, mg/dL, mean (SD)	215 (39)	204 (39)	198 (38)
HDL-C, mg/dL, mean (SD)	54 (15)	51 (16)	50 (15)
Serum albumin, mg/dL, mean (SD)	4.4 (0.3)	4.3 (0.3)	4.3 (0.3)
Triglyceride, mg/dL, mean (SD)	135 (81)	143 (88)	145 (108)
Life meaning, % impaired	4.0%	3.9%	6.7%
Satisfaction, % impaired	2.8%	2.2%	3.7%
Well-being, % impaired	1.7%	1.6%	3.1%

BMI, body mass index; CVD, cardiovascular disease; HDL-C, high density lipoprotein-cholesterol; HbA1c, glycated hemoglobin; SD, standard deviation

[a] CVD related treatment stands for receiving any blood pressure/glucose/lipid lowering medication therapy.

eTable 3. Baseline characteristics for 2005 follow-up participants

	Never smoking	Past smoking	Current smoking
n	2,780	636	1,186
Age, years, mean (SD)	63 (9)	65 (9)	62 (8)
Gender, male, %	16%	88%	83%
BMI, kg/m^2, mean (SD)	23 (3)	23 (3)	23 (3)
Systolic Blood Pressure, mm Hg, mean (SD)	142 (20)	144 (20)	144 (21)
CVD treatment, %[a]	28%	33%	23%
Drinking, %	10%	46%	53%
Salt preference, % prefer salty	47%	52%	62%
HbA1c, mmol/L, mean (SD)	5.1 (0.8)	5.1 (0.8)	5.2 (0.8)
Total cholesterol, mg/dL, mean (SD)	215 (39)	204 (39)	198 (38)
HDL-C, mg/dL, mean (SD)	55 (15)	52 (15)	50 (15)
Serum albumin, mg/dL, mean (SD)	4.4 (0.3)	4.3 (0.3)	4.3 (0.3)
Triglyceride, mg/dL, mean (SD)	134 (81)	143 (89)	145 (108)
Life meaning, % impaired	3.2%	2.7%	4.3%
Satisfaction, % impaired	2.1%	1.1%	3.2%
Well-being, % impaired	1.1%	1.3%	2.2%

BMI, body mass index; CVD, cardiovascular disease; HDL-C, high density lipoprotein-cholesterol; HbA1c, glycated hemoglobin; SD, standard deviation

[a] CVD related treatment stands for receiving any blood pressure/glucose/lipid lowering medication therapy.

eTable 4. Baseline characteristics for 2012 follow-up participants

	Never smoking	Past smoking	Current smoking
n	3,011	631	1,360
Age, years, mean (SD)	58 (8)	61 (8)	58 (8)
Gender, male, %	16%	87%	81%
BMI, kg/m^2, mean (SD)	23 (3)	23 (3)	22 (3)
Systolic Blood Pressure, mm Hg, mean (SD)	139 (20)	142 (20)	141 (21)
CVD treatment, %[a]	23%	30%	25%
Drinking, %	11%	50%	56%
Salt preference, % prefer salty	48%	52%	63%
HbA1c, mmol/L, mean (SD)	5.0 (0.8)	5.1 (0.9)	5.1 (0.9)
Total cholesterol, mg/dL, mean (SD)	214 (38)	206 (37)	199 (39)
HDL-C, mg/dL, mean (SD)	55 (15)	51 (15)	51 (15)
Serum albumin, mg/dL, mean (SD)	4.4 (0.3)	4.4 (0.3)	4.4 (0.3)
Triglyceride, mg/dL, mean (SD)	134 (83)	148 (88)	150 (91)
Life meaning, % impaired	38%	36%	40%
Satisfaction, % impaired	8.2%	9.7%	7.4%
Well-being, % impaired	4.6%	4.8%	4.6%

BMI, body mass index; CVD, cardiovascular disease; HDL-C, high density lipoprotein-cholesterol; HbA1c, glycated hemoglobin; SD, standard deviation

[a] CVD related treatment stands for receiving any blood pressure/glucose/lipid lowering medication therapy.
